# Investigation of Particles and Gas Bubbles in Zr–0.8Sn–1Nb–0.3Fe Zr Alloys Irradiated by Krypton Ions

**DOI:** 10.3390/ma11102056

**Published:** 2018-10-22

**Authors:** Wenzhu Shen, Chenwei Liu, Penghui Lei, Guang Ran

**Affiliations:** 1 School of Materials and Engineering, Southwest Petroleum University, Chengdu 610500, Sichuan Province, China; wenzhupinger@163.com; 2 College of Energy, Xiamen University, Xiamen 361102, Fujian Province, China; asucba@163.com

**Keywords:** N36 Zr alloy, irradiation behavior, gas bubbles, amorphization, particle

## Abstract

Two types of Zr–0.8Sn–1Nb–0.3Fe Zr alloys were irradiated by krypton ions in the temperature range from 320 to 400 °C. The microstructure of the as-received alloys showed that the sizes of Zr crystals and (Zr, Nb)_2_Fe particles with face-centered cubic (FCC) structure increased from 3.9 μm to 6.0 μm and from 74.6 nm to 89.6 nm, respectively, after cold rolling and subsequent annealing. Kr^+^ irradiation-induced bubble formation in the Zr matrix was observed. The size of the gas bubbles increased with increasing ion fluence and irradiation temperature. An equation that related the bubble size, ion fluence, and temperature were established. Irradiation-induced amorphization of particles was observed and found to be related to the fabrication process and irradiation parameters. The particles in alloy #1 showed a higher irradiation tolerance than those in alloy #2. The threshold damage dose for the amorphization of particles in alloy #2 was 3.5 dpa at 320 °C and 4.9 dpa at 360 °C. The mechanisms for bubble growth and particle amorphization are also discussed.

## 1. Introduction

Zr alloys are widely used in nuclear reactors due to their excellent comprehensive properties and performance [1,2]. Due to the requirements of further enhancement of the properties and performance of Zr alloys [3], a series of new types of Zr alloys have been developed around the world, such as in China, Russia, Europe, and the USA [4,5,6,7]. An advanced tolerant fuel (ATF) project was proposed in China to develop Zr alloy fuel cladding with better comprehensive performance [8]. Improvements to the properties and performance of Zr alloys are mostly made by optimizing the manufacturing process and the chemical composition by changing the contents of alloying elements [9,10]. A N36 Zr alloy with Zr–1.0Sn–1.0Nb–0.3Fe which showed excellent out-of-reactor performance was developed in China [11]. Recently, two kinds of further-modified N36 Zr alloys with chemical composition Zr–0.8Sn–1.0Nb–0.3Fe were proposed to obtain better comprehensive performance. In this paper, the evolution behaviors of the particles and gas bubbles of these two kinds of modified N36 Zr alloys during krypton ion irradiation are fully studied.

The service environment of Zr alloys is very severe, especially in terms of particle irradiation such as X-rays, neutrons, transmutation products, and decay products. The transmutation reactions (n, α) and (n, p) generate He atoms and H atoms that will degrade service performance with displacement damage including embrittlement, hardening, and irradiation swelling [12]. Therefore, the irradiation behavior of newly developed Zr alloys needs to be investigated before their use in commercial nuclear reactors. Up to now, many studies have reported the irradiation results of Zr alloys [13,14,15,16,17]. Nonetheless, some questions related to gas bubble growth and particle amorphization still need to be studied and answered. For example, the threshold displacement damage value for particle amorphization in a Zr–Sn–Nb Zr alloy irradiated with Ni^+^ ions was reported to be about 5 dpa at 300 °C [14]. However, Sun [15] indicated that Zr(Fe,Cr,Nb)_2_ particles with a hexagonal close-packed (hcp) structure began to be amorphized after only 0.5 dpa, and the critical irradiation dose for this type of particle to be completely amorphized was 1.0 dpa at 310 °C. Zu [16] reported that the amorphization of Zr(Fe,Cr)_2_ particles in a Zr-4 alloy had not occurred under 2 MeV H^+^ ion irradiation at a dose of 7 dpa at 350 °C, but partial amorphization was observed after 5 dpa at 310 °C. However, they saw that the Zr(Fe,Cr)_2_ particles in Zr-4 alloys were completely amorphized by 600 keV Ne^+^ ion radiation at 1.2 dpa and 350 °C. The amorphization of the particles should be controlled not only by the damage dose but also by the cascade size which is related to ion mass and energy. Jin reported that the threshold damage dose for particle amorphization increased with increasing irradiation temperatures [17]. The amorphization mechanism of particles in Zr alloys still needs to be further studied, especially for new Zr alloys.

In the present work, two types of modified N36 Zr alloys were irradiated with 400 keV Kr^+^ ions at different ion fluences and temperatures. The evolution behavior of gas bubbles and particles was fully studied.

## 2. Experiment

The two types of Zr alloys with Zr–0.8Sn–1Nb–0.3Fe (wt. %) chemical composition used in this study. For sample #1, the fabrication process was as follows: First, a 50 g ingot was rolled to form a sheet. Then, β phase homogenizing treatment and water quenching was carried out on the sheet. Next, the sheet was hot rolled and then cold rolled. Finally, the sheet was annealed (recrystallization, δ ≈ 0.65 mm). Compared with sample #1, sample #2 had an extra intermediate annealing step and a second cold rolling step. Therefore, these two kinds of modified N36 Zr alloys have differences in their microstructure and properties.

The preparation process of samples with dimensions 10 × 3 × 2 mm used for krypton ion irradiation can be found in our previous paper [18]. The electrochemical polishing solution was a 10 vol. % perchloric acid ethanol solution. The process for the preparation of the TEM (transmission electron microscopy) samples from the as-received sheet is described in our previous paper [5], as well as that for the krypton ion irradiation and the preparation of the cross-sectional TEM samples from the irradiated bulk samples. In brief, two groups were formed for the krypton ion irradiation process: one group of samples was irradiated with 2.86 × 10^15^ Kr^+^/cm^2^ ion fluence at temperatures ranging from 320 to 400 °C, and another group of samples was irradiated at 360 °C with ion fluence up to 7.14 × 10^15^ Kr^+^/cm^2^. The samples of 10 × 3 × 2 mm in dimensions were pasted on the surface of stainless-steel stage with Φ180 mm by carbon paste. The beam spot size of krypton ions was approximately Φ30 mm. The temperature during krypton ion irradiation was adjusted by thermoelectric couple embedded the stainless-steel stage and the cooling water.

## 3. Results and Discussion

### 3.1. Microstructure of the As-Received Alloys

**Figure 1:**
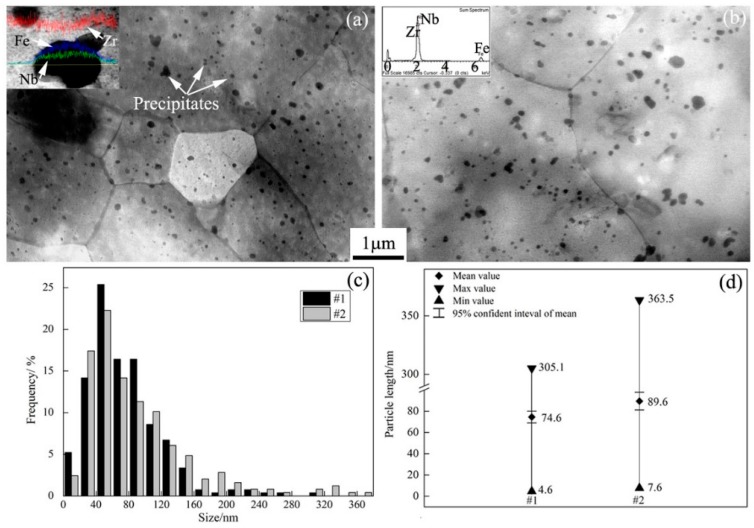
(**a**,**b**) STEM images showing the microstructure of the as-received #1 and #2 N36 Zr alloys, respectively; (**c**) the frequencies of particle lengths; (**d**) statistical results of the particle sizes.

STEM (scanning transmission electron microscope) images showing the microstructure of the as-received alloys #1 and #2 are displayed in Figure 1a,b, respectively. The crystal boundaries and many particles with black contrast can be observed in the Zr alloy matrix. Statistical analysis shows that the average crystal sizes of alloys #1 and #2 were 3.9 μm and 6.0 μm, respectively. Apparently, the extra process of intermediate annealing and a second cold rolling induced Zr crystal growth in alloy #2. The average crystal sizes of these two kinds of N36 Zr alloys are similar to those of the Zr alloys reported in Zhao’s paper [19] and Nikulina’s study [20].

The inset images in Figure 1a,b are line scanning and point measurements, respectively, of chemical elements in typical particles as found by EDS (energy-dispersion spectrum). The element peaks of Fe, Nb, and Zn can be found in the particles. Due to the easy dissolution of Sn atoms in the Zr matrix, it is difficult to detect Sn in the particles [21,22]. Thus, these particles should be the alloy compounds of Zr, Nb, and Fe elements in these two kinds of modified N36 Zr alloys.

Figure 1c shows the frequencies of particle lengths. The total percentages of particles ranging in size from 30 to 130 nm in Zr alloys #1 and #2 were 90% and 80%, respectively. The average sizes of particles in Zr alloys #1 and #2 were 74.6 nm and 89.6 nm, respectively. The 95% confidence intervals of the particle lengths in Zr alloys #1 and #2 were [69.1~80.1] nm and [81.3~97.9] nm, respectively, as shown in Figure 1d. The average size and the 95% confidence interval of the particles in alloy #1 are both obviously smaller than those in alloy #2. However, the sizes of the particles in these two kinds of modified N36 Zr alloy are both smaller than the 100~200 nm particle size reported for a Zr alloy by Nikulina [20].

**Figure 2:**
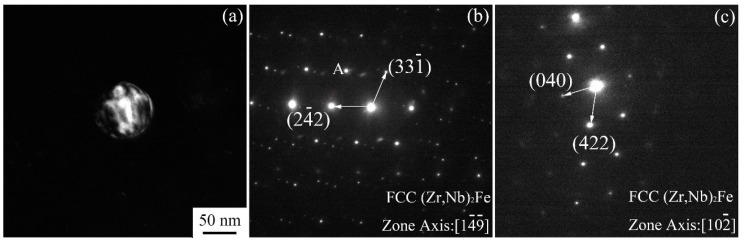
TEM dark field image and corresponding selected area electron diffraction patterns of a particle in Zr alloy #2. (**a**) TEM dark field image from the ‘A’ spot of the SAED (selected area electron diffraction) pattern in (**b**); (**b**,**c**) SAED patterns from different crystal zone axes.

A dark field TEM image and corresponding SAED (selected area electron diffraction) patterns of a particle in as-received alloy #2 are shown in Figure 2. The dark field image of a particle of about 75 nm shown in Figure 2a was obtained using diffraction spot A in Figure 2b. The SAED patterns in Figure 2b,c were both derived from the particle in Figure 2a but from different zone axes. According to the SAED and EDS test results, the particle was determined to be (Zr,Nb)_2_Fe with a face-centered cubic crystal structure according to the JCPDS standard cards. The lattice constant *a* was found to be 1.21 nm by indexing and calculating the diffraction patterns. The diffraction zone axes are [1 −4 −9] and [1 0 −2] in the SAED patterns in Figure 2b,c, respectively. The crystal structure characteristics of particles in alloy #1 were the same as those in alloy #2.

### 3.2. Irradiation Behavior of Particles

**Figure 3:**
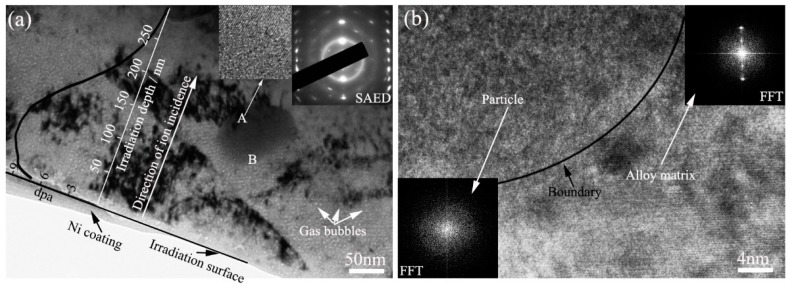
TEM bright field images showing the microstructure of Zr alloy #1 irradiated with 2.86 × 10^15^ Kr^+^/cm^2^ fluence at 320 °C. (**a**) Under-focused bright field image; (**b**) High-resolution TEM (HRTEM) image showing the boundary area between a particle and the Zr alloy matrix.

Figure 3 displays TEM images showing the microstructure of Zr alloy #1 irradiated with 2.86 × 10^15^ Kr^+^/cm^2^ fluence at 320 °C. The irradiation surface and the damage depths, nickel coating, and incident direction of Kr^+^ ions are drawn in the TEM image in Figure 3a, as well as SRIM (The Stopping and Range of Ions in Matter) simulation results on the relationship between displacement damage and irradiation depth. The total penetration depth of 400 keV Kr^+^ ions is about 350 nm. The peak displacement damage is 10 dpa at about 70 nm depth.

A particle of about 100 nm in size, observed at location A of Figure 3a in the Zr matrix, was analyzed in detail. The particle location was 187 nm below the irradiation surface and the displacement damage there was about 3.5 dpa. High-resolution TEM (HRTEM) images of location A are shown in Figure 3a. The long-range order of the crystal structure of the matrix and amorphous structure of the particle are both observed in alloy #1, which indicates that the particle was partially changed to an amorphous state after irradiation and 3.5 dpa displacement damage at 320 °C. The SAED pattern from location B in the particle is shown at the top-right corner of Figure 3a and contains the amorphous diffraction halo and crystal diffraction spots that should come from the particle and the Zr matrix, respectively. An HRTEM image of location B shows the area between the particle and the Zr matrix in Figure 3b. The long-range order of the crystal structure is observed in the Zr matrix, indicating that Kr^+^ irradiation does not induce amorphization of the Zr crystal. However, an amorphous structure is observed in the particles. The Fast Fourier Transform (FFT) patterns of particles and the Zr matrix are inserted at the lower-left corner and at the top-right corner of Figure 3b and indicate the amorphous diffraction character and crystal diffraction character, respectively. They further prove that the particle is amorphous and the Zr matrix is crystalline. The SRIM simulation results show that the displacement damage at location B is about 7.5 dpa. Therefore, it can be concluded that the threshold value of displacement damage for a particle to transform to an amorphous state in Zr alloy #1 is an average value of 3.5 dpa at 320 °C.

**Figure 4:**
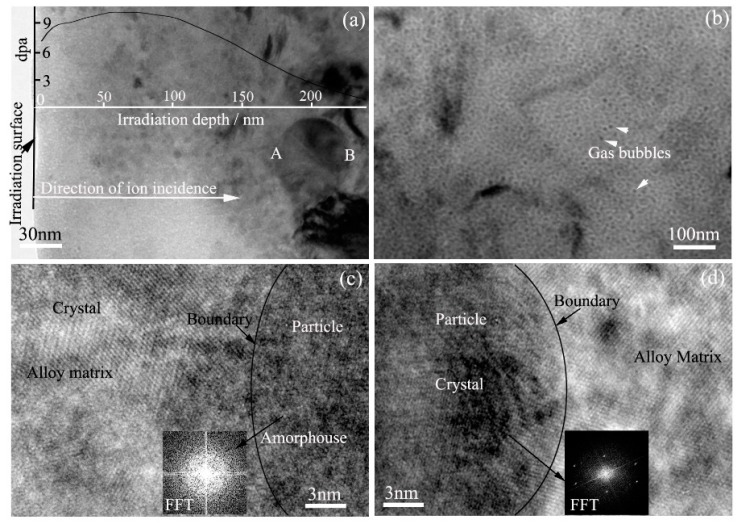
TEM bright field images showing the microstructure of Zr alloy #2 irradiated with 2.86 × 10^15^ Kr^+^/cm^2^ fluence at 320 °C. (**a**) Low magnification of an under-focused TEM image; (**b**) Over-focused TEM image showing gas bubbles; (**c**,d) HRTEM images showing the boundary area between a particle and the Zr matrix.

TEM bright field images of Zr alloy #2 irradiated with 2.86 × 10^15^ Kr^+^/cm^2^ fluence at 320 °C are shown in Figure 4. Particles of about 60 nm in size are distributed in the irradiation area as shown in Figure 4a. Location A and location B in the particle are located at irradiation depths of 170 nm and 230 nm, respectively. According to SRIM simulation results, the levels of displacement damage at location A and location B are about 4 dpa and 2 dpa, respectively. High-resolution TEM images of location A and location B are shown in Figure 4c,d, respectively. The interface can be observed between the particle and the Zr matrix. In Figure 4c, long-range order in the crystal structure in the Zr matrix and an amorphous structure in the particle are observed, indicating that the particle is amorphous and the Zr matrix is crystalline. The inset image in Figure 4c is the FFT pattern of the particle, which further demonstrates that the particle is amorphous. Therefore, 4 dpa displacement damage at 320 °C induces particle state change from crystal to amorphous in Zr alloy #2.

However, in Figure 4d, both the Zr matrix and the particle are long-range-ordered crystal structures, which indicates that both of them are crystal structures. The FFT pattern of the particle in Figure 4d further proves that conclusion. Therefore, 2 dpa displacement damage at 320 °C does not induce particle change from crystal to amorphous. It can be concluded that the threshold value of displacement damage inducing particle change from crystal to amorphous in Zr alloy #2 is an average value of 3.2 dpa at 320 °C.

**Figure 5:**
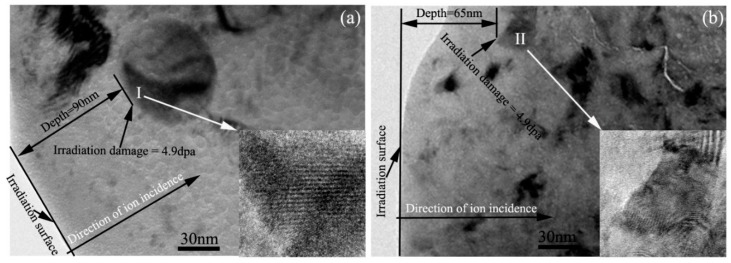
TEM bright field images showing the microstructure of the Zr alloys irradiated with 1.43 × 10^15^ Kr^+^/cm^2^ fluence at 360 °C. (**a**) Zr alloy #1; (**b**) Zr alloy #2.

Figure 5a,b are TEM bright field images showing the microstructure of Zr alloys #1 and #2, respectively, irradiated with 1.43 × 10^15^ Kr^+^/cm^2^ fluence at 360 °C. The SRIM results show that the peak displacement damage is 5 dpa at 70 nm depth. Location ‘I’ in Figure 5a and location ‘II’ in Figure 5b are located at 90 nm and 65 nm irradiation depths, respectively. The displacement damage values at location ‘I’ and location ‘II’ are both about 4.9 dpa. High-resolution TEM images of location ‘I’ and location ‘II’ are shown in the inserted images located at the bottom right of Figure 5a,b, respectively. The long-range order of the crystal structure is observed in the particle in Zr alloy #1, indicating that the particle still has a crystal structure even though it was irradiated by 4.9 dpa displacement damage at 360 °C. However, we only can observe long-range order of the crystal structure at the edge of the particle in Zr alloy #2 and a partial region is changed to an amorphous structure, indicating that the particle partially changed to amorphous after irradiation with 4.9 dpa displacement damage at 360 °C. Therefore, the particles in Zr alloy #1 have higher irradiation resistance ability than those in Zr alloy #2.

As mentioned above, the particles in Zr alloy #1 have a higher amorphous threshold value than do those in Zr alloy #2. The threshold values of particle change from crystal to amorphous are 3.2 dpa at 320 °C and 4.9 dpa at 360 °C in Zr alloy #2; correspondingly, the threshold value is 3.5 dpa at 320 °C in Zr alloy #1. It can be seen that the irradiation temperature is a key factor for particle amorphization. When the rate of irradiation-induced amorphization is higher than that of thermally activated annealing, particles will transform from crystal to amorphous. The values in the current study results are higher than those reported in Zu’s and Sun’s studies [15,16]. Zu [16] reported that Zr(Fe,Cr)_2_ particles in the Zr-4 alloy were completely amorphized after 600 keV Ne^+^ ion irradiation up to 1.2 dpa damage at 350 °C. Sun [15] reported that Zr(Fe,Cr,Nb)_2_ particles were amorphized by 500 keV Ne^+^ ion irradiation with only 1.0 dpa irradiation damage at 310 °C.

### 3.3. Irradiation Behavior of Gas Bubbles

Figure 3 and Figure 4 show the microstructures of Zr alloys #1 and #2, respectively, irradiated with 2.86 × 10^15^ Kr^+^/cm^2^ fluence at 320 °C. It can be seen in Figure 3a and Figure 4b that a large number of gas bubbles formed in the Zr matrix. The morphology and distribution characteristics of the gas bubbles are similar in these two Zr alloys. The statistical results show that the average sizes of the bubbles in Zr alloys #1 and #2 were about 2.5 nm and 2.76 nm, respectively. Faulkner [23] reported that void growth was observed to occur after irradiation in a sample pre-injected with 100 ppm helium. The Kr^+^ ion concentration in Faulkner’s experiment was notably smaller than that in our work. Zr alloys #1 and #2 irradiated with 2.86 × 10^15^ Kr^+^/cm^2^ fluence at 360 °C and 400 °C were also investigated and analyzed in the present work. Similarly, many gas bubbles were distributed in the irradiation area. The average sizes of the bubbles in Zr alloy #1 were 2.75 and 3.19 nm at 360 °C and 400 °C, respectively. Correspondingly, the values were 3.11 and 3.74 nm, respectively, in Zr alloy #2.

**Figure 6:**
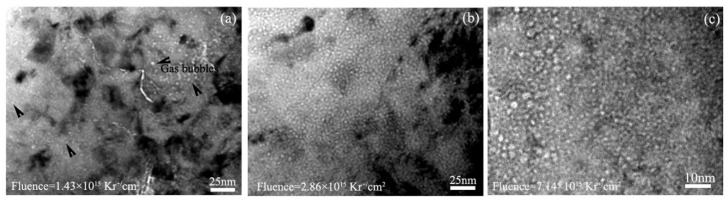
TEM bright field images showing the morphologies of Zr alloy #2 irradiated with (**a**) 1.43 × 10^15^, (**b**) 2.86 × 10^15^, and (**c**) 7.14 × 10^15^ Kr^+^/cm^2^ fluence at 360 °C.

Figure 6 displays under-focused bright field TEM images showing the morphologies of the bubbles in Zr alloy #2 irradiated with different krypton ion fluences at 360 °C. The statistical results show that the average sizes of the bubbles were 2.98, 3.11, and 3.7 nm after irradiation with 1.43 × 10^15^, 2.86 × 10^15^, and 7.14 × 10^15^ Kr^+^/cm^2^, respectively. Correspondingly, the bubble sizes were 2.47 nm, 2.75 nm, and 3.55 nm in Zr alloy #1. Obviously, the average bubble size increases with increasing krypton ion fluence. Moreover, at same ion fluence, the average bubble size in Zr alloy #2 is larger than that in Zr alloy #1, which indicates that the swelling tendency of Zr alloy #2 is larger than that of Zr alloy #1. This difference is due to the different fabrication processes which change the solid solubility of alloying elements in the Zr matrix; this has an important influence on defect diffusion [24] and bubble formation [25] in Zr alloys.

Krypton ions were continuously implanted into the modified N36 Zr alloys at a given temperature. Therefore, the growth of gas bubbles should be controlled by the newly injected gas atoms, the newly created vacancies, the diffusion of undissolved gas atoms and vacancies, and the migration of small bubbles [26,27,28,29]. Consequently, for the same Zr alloy irradiated at a given ion fluence, the different sizes of gas bubbles are due to the effect of different temperatures providing different levels of kinetic energy for the movement of gas atoms, vacancies, and bubbles [30]. The higher the irradiation temperature applied, the larger the average gas bubble size will be. For different kinds of Zr alloys irradiated at the same ion fluence and irradiation temperature, the different sizes of gas bubbles are due to the differences in microstructure and the amount of microalloy in the Zr matrix providing different levels of resistance to defect movement. The size of krypton bubbles in Zr alloy #2 is larger than that in Zr alloy #1 due to the extra intermediate annealing and second cold rolling step in the fabrication of Zr alloy #2 inducing the dissolution of microalloy elements out of the Zr matrix.

The equation *D_b_* = *f exp*(−*E_r_*/*kTF*), as shown in reference [5], can be used to describe the relationship between *D_b_* (the average diameter of gas bubbles in nanometers) and *E_r_* (the effective activation energy of coarsening, *E_r_* = 0.315 eV [31]). In the above equation, *k* and *T* are the Boltzmann constant and irradiation temperature in degrees kelvin, respectively; *F* and *f* are the displacement damage in dpa and functional coefficient, respectively. *f* is related with the irradiation temperature, displacement damage, and characteristics of the material itself. The size of krypton bubbles in the present work is shown in Table 1. The average sizes of gas bubbles increase with increasing temperature and displacement damage.

The *f* values are also listed in Table 1. According to the data, equations giving the relationship between *f* and experiment parameters such as irradiation temperature and displacement damage can be obtained for a specific experimental condition.

**Figure 7:**
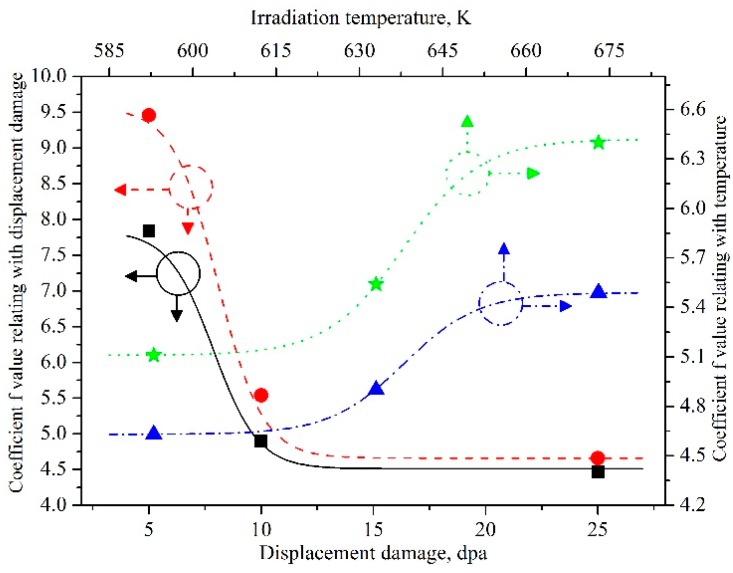
The functional coefficient *f* vs. experiment parameters during the growth of krypton bubbles. The solid line with black color represents *f* vs. displacement damage for Zr alloy #1; the dash line with red color represents *f* vs. displacement damage for Zr alloy #2; the dot line with blue color represents *f* vs. irradiation temperature for Zr alloy #1; the dot line with green color represents *f* vs. irradiation temperature for Zr alloy #2.

According to Figure 7, when the irradiation temperature is constant, the best-fitting equation can be obtained as follows:

for Zr alloy #1:
  *f* = 4.51 + 3.33/(1 + exp(*F* − 7.87)), (1)
for Zr alloy #2:
  *f* = 4.66 + 4.91/(1 + exp(*F* − 8.06)). (2)

When displacement damage is constant, the equations are:

for Zr alloy #1:
  *f* = 4.63 + 0.86/(1 + 10^(0.0625×(638−*T*))^), (3)
for Zr alloy #2:
  *f* = 5.11 + 1.31/(1 + 10^(0.0625×(638−*T*))^). (4)

Using Equations (1)–(4), it is possible to calculate the size of gas bubbles in the Zr alloys at specified values of irradiation temperature and displacement damage.

## 4. Conclusions

Two types of modified N36 Zr alloy with the chemical composition Zr–0.8Sn–1Nb–0.3Fe Zr were irradiated using 400 keV krypton ions from 5 dpa to 25 dpa at 320 °C to 400 °C. The microstructures of the as-received Zr alloys and cross-sectional irradiation samples were analyzed by transmission electron microscopy. The main results were as follows:

(1) The sizes of the Zr crystal and its particles became large after additional cold rolling and annealing steps. The sizes of the crystal and particles grew from 3.9 μm to 6.0 μm and from 74.6 nm to 89.6 nm, respectively. Most particles in the Zr matrix were (Zr,Nb)_2_Fe compounds with an FCC structure.
(2) Amorphization of particles was observed in both irradiated alloys, and the amorphization dose was significantly influenced by the fabrication process and irradiation parameters. The particles in alloy #1 had higher irradiation tolerance than did those in alloy #2. The threshold values of displacement damage inducing particle change from crystal to amorphous were 3.2 dpa and 3.5 dpa in alloys #1 and #2, respectively, at 320 °C. The threshold value was 4.9 dpa in alloy #2 at 360 °C. At the corresponding displacement damage and irradiation temperature, particles retained a crystal structure in Zr alloy #1.
(3) Kr^+^ irradiation induced krypton bubble formation in the Zr alloy matrix. The size of the krypton bubbles increased with increasing displacement damage and irradiation temperature. The average size of gas bubbles in Zr alloy #2 was larger than that in Zr alloy #1 under the same experimental conditions. Equations relating bubble size with experiment parameters were obtained from the experiment data.

## Figures and Tables

**Table 1:** The sizes of gas bubbles and coefficient *f* values.

	Experiment Conditions	Irradiation Temperature, *T*, 360 °C			Displacement Damage, *F*, 10 dpa		
Samples		5 dpa	10 dpa	25 dpa	320 °C	360 °C	400 °C
Bubble Size, nm	Zr alloy #1	2.5 ± 0.03	2.8 ± 0.03	3.6 ± 0.03	2.5 ± 0.03	2.8 ± 0.03	3.2 ± 0.03
	Zr alloy #2	3.0 ± 0.03	3.1 ± 0.03	3.7 ± 0.03	2.8 ± 0.03	3.1 ± 0.03	3.7 ± 0.03
*f Value*	Zr alloy #1	7.84	4.9	4.47	4.63	4.9	5.49
	Zr alloy #2	9.46	5.54	4.66	5.11	5.54	6.44
